# Association between ambulatory blood pressure monitoring patterns with cognitive function and risk of dementia: a systematic review and meta-analysis

**DOI:** 10.1007/s40520-023-02361-7

**Published:** 2023-03-30

**Authors:** Maria Gavriilaki, Panagiota Anyfanti, Konstantinos Mastrogiannis, Eleni Gavriilaki, Antonios Lazaridis, Vasilios Kimiskidis, Eugenia Gkaliagkousi

**Affiliations:** 1 1st Department of Neurology, AHEPA University Hospital, School of Medicine, Aristotle University of Thessaloniki, Thessaloniki, Greece; 2 3rd Department of Internal Medicine, Papageorgiou Hospital, Aristotle University of Thessaloniki, Ring Road Nea Efkarpia, 56429 Thessaloniki, Greece; 3 Hematology Department, G. Papanikolaou Hospital, Thessaloniki, Greece

**Keywords:** Meta-analysis, Ambulatory blood pressure measurement, Dipping patterns, Cognitive dysfunction, Dementia, Neuropsychological tests

## Abstract

**Background:**

The objective of this systematic review and meta-analysis is to investigate whether nocturnal blood pressure fall, expressed by dipping patterns according to 24 h ambulatory blood pressure monitoring (ABPM), is associated with abnormal cognitive function (cognitive impairment or dementia).

**Methods:**

We systematically searched PubMed, Embase, and Cochrane databases to identify original articles through December 2022. We included any study with at least ten participants reporting on all-cause dementia or cognitive impairment incidence (primary outcome) or validated cognitive tests (secondary outcome) among ABPM patterns. We assessed risk of bias using Newcastle–Ottawa Quality Assessment Scale. We pooled odds ratios (OR) and standardized mean differences (SMD) using random-effect models for primary and secondary outcome, respectively.

**Results:**

In the qualitative synthesis, 28 studies examining 7595 patients were included. The pooled analysis of 18 studies showed that dippers had a 51% [OR 0.49(0.35–0.69)] lower risk of abnormal cognitive function and a 63% [OR 0.37(0.23–0.61)] lower risk of dementia alone, compared to non-dippers. Reverse dippers presented an up to sixfold higher risk [OR 6.06(3.15–11.64)] of abnormal cognitive function compared to dippers and an almost twofold higher risk [OR 1.81(1.26–2.6)] compared to non-dippers. Reverse dippers performed worse in global function neuropsychological tests compared with both dippers [SMD − 0.66(− 0.93 to − 0.39)] and non-dippers [SMD − 0.35(− 0.53 to − 0.16)].

**Conclusion:**

Dysregulation of the normal circadian BP rhythm, specifically non-dipping and reverse dipping is associated with abnormal cognitive function. Further studies are required to determine potential underlying mechanisms and possible prognostic or therapeutic implications.

**Protocol registration:**

PROSPERO database (ID: CRD42022310384).

**Supplementary Information:**

The online version contains supplementary material available at 10.1007/s40520-023-02361-7.

## Introduction

Dementia is an important public health issue with major socioeconomic implications and resource allocation in healthcare [1]. The global burden of dementia is anticipated to undergo a huge growth from 57.4 million cases in 2019 to 152.8 million cases in 2050, mainly attributed to increases in population growth and ageing [2]. Alzheimer’s disease is the leading cause of dementia followed by vascular dementia, although both types may frequently overlap with mixed pathologies among dementia cases [3]. Despite continuously increasing investment into research, no effective disease-modifying therapies currently exist [4]. Therefore, interventions targeting modifiable risk factors emerge as extremely important for alleviating the global burden of the disease, which could theoretically prevent or delay up to 40% of dementia prevalence [5].

Hypertension is a highly prevalent condition that constitutes a major risk factor of dementia [6]. Hypertension-induced cerebrovascular changes include a wide range of complex pathophysiological processes leading to divergent clinical outcomes from the brain [7]. However, much is still unknown about which and how hypertension-related mechanisms exert a detrimental effect on cognitive function and promote Alzheimer’s and vascular dementia in humans [8]. For instance, age of hypertension diagnosis, hypertension chronicity, and intensity of treatment have all been recognized as factors affecting cognitive function, although with regard to the latter, optimal blood pressure (BP) levels to preserve cognitive function remain controversial [9, 10].

Compared to conventional office BP measurements, 24 h ambulatory BP monitoring (ABPM) unveils the physiological variance of BP throughout both daytime and nighttime and is superior in predicting hypertension-mediated organ damage and future cardiovascular events [11–13]. Physiologically, nighttime BP values are expected to normally drop by 10–20% compared to daytime values, which enables the classification of patients as “dippers” (≥ 10%) and “non-dippers” (< 10%) [14]. Non-dipping pattern is encountered in a significant portion of the general hypertensive population (20–40%) [15, 16], and is considered a strong indicator of hypertension-mediated organ damage and increased cardiovascular morbidity and mortality [17–19]. The worst incidence of fatal and non-fatal cardiovascular events has been reported among the so called “reverse dippers” or “risers” (non-dippers with nighttime BP dipping < 0%, owing to even higher nighttime compared to daytime BP) [20]. Paradoxically, there is also some evidence of increased risk among patients who are characterized as extreme dippers (nighttime BP drop ≥ 20%) [19]. However, the prognostic value of dipping categories may vary according to the study population and the outcomes of interest [21]. For instance, we have recently shown in a systematic review and meta-analysis that reverse dipping, rather than the non-dipping phenomenon in general, is a significant risk factor for cardiovascular events in untreated hypertensives [22].

With regard to dementia and cognitive impairment, cross-sectional investigations of ABPM and cognition have produced inconsistent results [23, 24]. Some of the relevant studies suggest that dipping status was associated with increased risk of dementia or cognitive impairment [25, 26], while others failed to prove any association [27, 28]. The objective of this systematic review and meta-analysis is to elucidate whether different circadian BP patterns, determined by 24 h ABPM, represent a risk factor of dementia or cognitive impairment incidence.

## Methods

Study's protocol was registered at PROSPERO database (ID: CRD42022310384). This systematic review and meta‐analysis is reported in accordance with the Preferred Reporting Items for Systematic reviews and Meta‐analysis (PRISMA) and Meta-analysis of Observational Studies in Epidemiology (MOOSE) guidelines [29, 30] (online‐only supplementary Table 1–2).

### Eligibility criteria

We included any original (randomized controlled trial, case–control, cross-sectional or cohort) study examining any population (with more than ten individuals) who underwent both 24 h ABPM and cognitive function assessment. All eligible studies reported effect estimates on validated cognitive tests or incidence of all-cause dementia or cognitive impairment among ABPM patterns. Dipping patterns were defined according to current hypertension guidelines [31]. Thus, the dipping patterns were classified as follows:

(1) Into the traditional dipping/non-dipping two groups; defined by systolic night-to-day ratio (SBP-NDR) ≤ 0.9 and > 0.9 or by dipping ratio [100 × (daytime − nighttime SBP)/daytime SBP] ≥ 10% and < 10% blood pressure (BP) fall, respectively, or.

(2) Into four groups; defined by SBP-NDR as extreme dippers < 0.8, dippers ≥ 0.80 to < 0.90, non-dippers ≥ 0.90 to < 1.00, and reverse dippers ≥ 1.00 or alternatively, by dipping ratio as dippers patients with ≥ 10% fall of daytime BP values, non-dippers < 10%, extreme dippers ≥ 20%, and reverse dippers or risers < 0%. We excluded studies examining the effect of an intervention (such as exercise or antihypertensive treatment) on dipping patterns and cognitive function.

### Information sources and search

We searched electronic databases, including MEDLINE (via PubMed), EMBASE (via Ovid), and the Cochrane Library, from inception through December 2022. No filters were used to limit literature search by records’ year of publication. A basic search strategy was developed for MEDLINE as published in the protocol of the study and modified accordingly for other databases (Supplementary material II). We also hand searched ClinicalTrials.gov site and the reference lists of both identified studies and relevant reviews. At last, authors were contacted to retrieve any unpublished data.

### Data extraction (selection and coding)

Records retrieved from literature search were imported into a reference management software (EndNote X7). Titles and abstracts were screened after duplicate removal by two reviewers (MG, EG) independently. Then two reviewers (MG, KM) perused full texts of eligible studies and independently extracted data using an excel form. Any conflicts were resolved by consensus discussion and consultation with a senior reviewer (PA). When multiple reports were published from the same cohort examining the same outcomes, we included in the qualitative analysis the one providing the most complete data to minimize duplication or overlapping samples.

We extracted data regarding study identification, design, funding and population characteristics, ABPM parameters and patterns, neuropsychological tests, cognitive impairment or/and dementia definitions along with the effect sizes of the aforementioned outcomes among dipping groups.

### Risk of bias assessment in individual and across studies

The quality of each study included in the analysis was assessed by two independent reviewers (MG, PA) using Newcastle–Ottawa Quality Assessment Scale (NOS) for cross-sectional or cohort studies and the revised Cochrane Collaboration’s Risk of Bias tool for randomized controlled trials [32, 33]. NOS comprises three question domains, each of which addresses a different type of bias (cohorts’ selection; comparability of cohorts; outcome assessment), with multiple possible answers some of which provide a star. According to the stars collected in each domain, the studies were stratified as high, fair, or low risk of bias. A study was deemed of high risk of bias when it was graded with none or one star in selection domain or none stars in comparability domain or none or one star in outcome domain. We examined funnel plot asymmetry to evaluate publication bias despite considerable limitations of all methods [34]. We also used the Grading of Recommendations, Assessment, Development and Evaluations (GRADE) approach to evaluate the certainty in the body of evidence [35]. Despite the lack of specific GRADE guidelines for observational studies, we assigned each pooled outcome a high degree of certainty and then downgraded or upgraded according to the certainty of evidence (risk of bias, inconsistency, indirectness, imprecision, publication bias, large effect or plausible confounding).

### Summary measures and synthesis of results

The primary outcome of interest was the risk of abnormal cognitive function (defined as the sum of dementia of any type or cognitive impairment cases) among dipping patterns. The secondary outcome was to compare cognitive test scores among dipping patterns. We focused on five cognitive domains: global function, attention/working memory, delayed memory, executive function, and processing speed. To be included in the quantitative analysis, a cognitive domain had to be examined by three or more studies to draw a reliable conclusion [36].

We performed a quantitative analysis for each outcome whenever possible using Review Manager Software version 5 (Cochrane Collaboration). We applied random-effects model using the inverse variance method. For continuous outcomes, we calculated standardized mean differences (SMD) and odds ratios (OR) for dichotomous outcomes; both presented with 95% confidence intervals (CIs) with a significance level set at *p* < 0.05. We tested heterogeneity among studies using *I*^2^ statistics [37]. When *I*^2^ exceeded 50%, the heterogeneity was considered substantial and a sensitivity or subgroup analysis was performed whenever possible [38].

We conducted a sensitivity analysis by excluding studies presenting high risk of bias whenever heterogeneity was high. Subgroup analyses were stratified according to dipping patterns [into four dipping (dippers, extreme dippers, non-dippers or reverse dippers) groups defined according to current hypertension guidelines previously described] and cognitive impairment subtypes as defined by the original records (mild cognitive impairment and dementia) [31]. We also performed a pre-planned subgroup analysis according to dementia types.

## Results

### Study selection—flow diagram

**Fig. 1:**
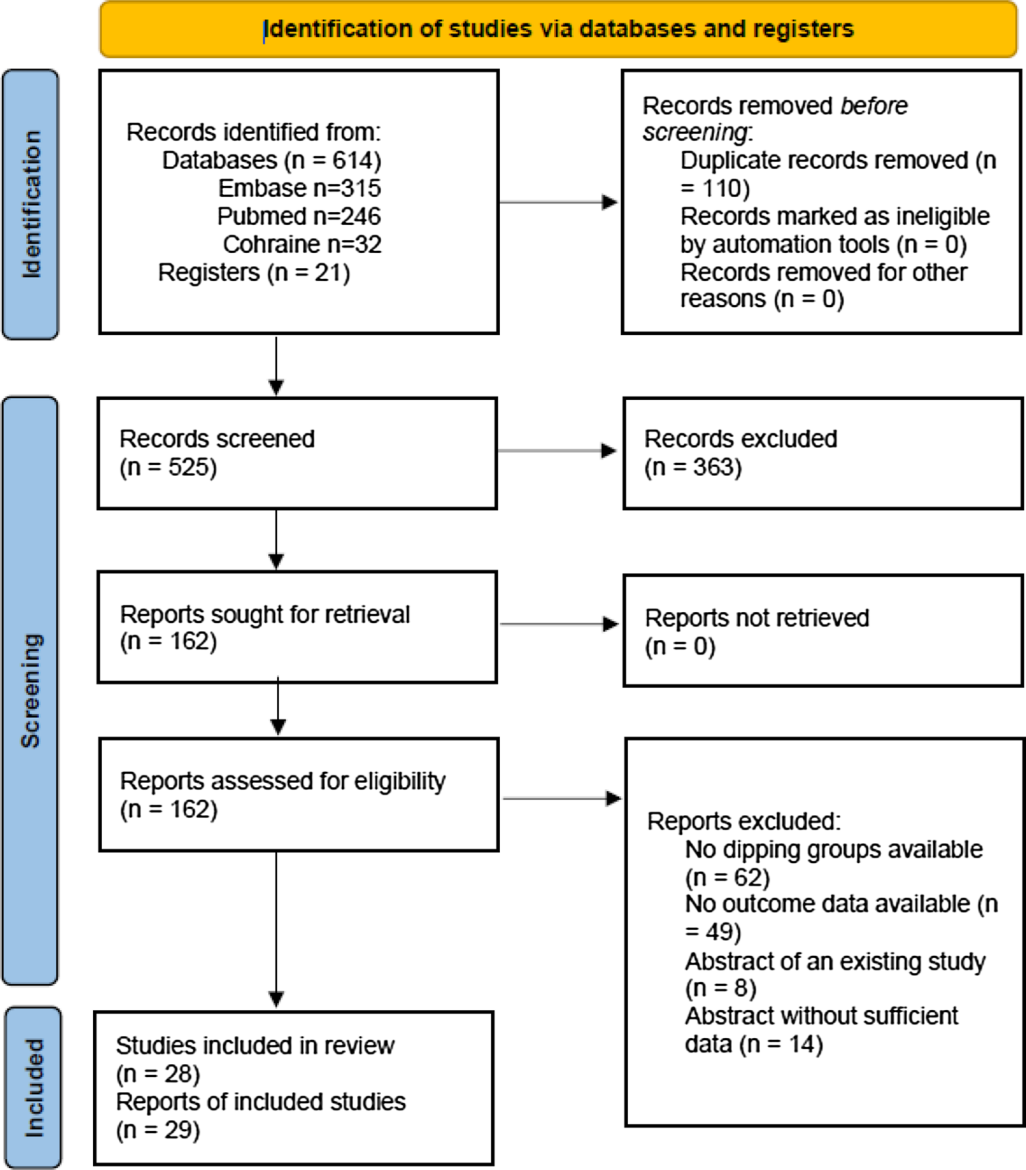
PRISMA 2020 flow diagram

Literature search yielded 525 records as shown at PRISMA flow diagram (Fig. 1). We included in the qualitative synthesis 29 reports of the 28 eligible studies examining 7595 patients. White et al. published two reports of different designs examining the relationship between 24 h ABPM and cognitive outcomes (executive function and processing speed) in patients 75 years of age and older [39, 40]. These two reports were merged in the qualitative analysis as predetermined in the methods. Several studies examining cognitive function among ABPM patterns were excluded because of lack of any effect estimates report, a predetermined exclusion criterion [41–43]. Tables 1 and 2 present the characteristics and risk of bias assessment of included studies.

**Table 1:** Summary of studies reporting on circadian blood pressure patterns and risk of cognitive impairment and/or dementia

Identity	*N* (% Males)		Population characteristics	Age [mean ± SD (range), years]	Circadian blood pressure patterns	Definition/Criteria used for the diagnosis of cognitive decline/dementia	Cognitive Outcome	NOS score
*Cross-sectional studies*								
Chen 2013 [44]	318 (54%)		Without hypertension or cerebrovascular history	na (65–85)	D, ED, ND, RD	AD: NINCDS-ADRDA	na	Low risk
Daniela 2021 [45]	90 (48.9%)		Without cerebrovascular history	74.7 ± 4.6	D, ED, ND, RD	AD: NINCDS-ADRDA, VaD: NINDS-AIREN/mHIS	MMSE, FAB	High risk
Guo 2010 [46]	144 (34%)		Aged ≥ 50 years without dementia, treated hypertension or cerebrovascular history	68 ± 7	D, ED, ND, RD	na	JMCIS	Low risk
Kececi 2016 [47]	91 (23%)		Aged ≥ 60 years hypertensive patients without comorbid diseases	71.9 ± 7.7	D, ND	sMMT < 24	sMMT	Fair
Kim 2009 [48]	109 (58%)		na	69.9 ± 4.1 (64–76)	D, ED, ND, RD	VaD: CDR ≥ 1 + IADL ≥ 8 + HI ≥ 5, MCI: CDR 0.5 + I-ADL < 8 + HI ≥ 5	Seoul Neuropsychological Screening Battery	Low risk
Komori 2016 [49]	444 (61.5%)		Hospitalized heart failure patients without cardiovascular or cerebrovascular history	68 ± 13	D, ND, RD	MCI: MMSE 21–26, Dementia: MMSE ≤ 21	MMSE	Low risk
Li 2017	108 (52.8%)		Patients with cerebral small vessel disease	67.7 ± 14.2 (45–78)	D, ED, ND, RD	MoCA < 23	MoCA	High risk
Okuno 2003 [51]	204 (31%)		Elderly subjects without cerebrovascular history	75.2 ± 7.2 (60–90)	D, ED, ND	MMSE ≤ 23	MMSE	Low risk
Paganini-Hill 2019 [26]	121 (37%)		Individuals aged ≥ 90 years	93.2 ± 0.3	D, ED, ND, RD	Diagnostic and Statistical Manual of Mental Disorders criteria	MMSE, 3MS	Low risk
Shim 2021 [27]	174 (31.6%)		Older adults with cognitive complaints	75.36 ± 7.13	D, ND, RD	MCI: Petersen’s criteria, AD: NINCDS-ADRDA	MMSE	Low risk
Tadic 2019 [52]	471 (53%)		Individuals randomly selected from the residents in Monza	na (25–74)	D, ND	MCI: MMSE 21‐24, moderate CI: MMSE 10‐20	MMSE	High risk
Tanaka 2018 [53]	137 (46%)		Parkinson’s disease patients	64.1 ± 10.5	D, ED, ND, RD	PDD: MDS Task Force diagnostic criteria	MMSE, HDS-R	Low risk
Xing 2021 [54]	305 (69%)		Elderly participants without renal disease	80.6 ± 7.6	D, ND, RD	MMSE < 27	MMSE	High risk
Yamamoto 2005 [55]	200 (61%)		Patients with first-time symptomatic lacunar infarct without cardiovascular history ^±^	68.8 ± 9.3 (43–87)	D, ND, RD	Cognitive impairment and dementia were diagnosed if there was both a CDR score ≥ 0.5 and a HDSR score ≤ 24	CDR, HDS-R	Low risk
Yamamoto 2011 [56]	224 (59.8%)		Patients with first-time symptomatic lacunar infarct^f^	69.8 ± 9.34	D, ND, RD	MCI: MMSE 25–27, dementia: MMSE ≤ 24	MMSE	Low risk
*Cohort studies*								
Cani 2022 [66]	30 (70%)	Patients with at least 5-year disease duration of cardiovascular autonomic failure and normal neurologic examination		66.8 ± 8.4	D, ND	Cognitive impairment was defined as an abnormal score on at least one test of the Neuropsychological assessment	MMSE, FR BMDB, RAVLT IR, RAVLT DR, Barrage test, IVM, SVAT, Stroop test, FP, FS, CD, PC	Fair
Tan 2021	1608 (100%)		Men without history of cognitive impairment or stroke	73.5 ± 3.3	D, ND, RD	Dementia: Diagnostic and Statistical Manual of Mental Disorders criteria, VaD: Alzheimer’s Disease Diagnostic and Treatment Center core criteria, AD: NINCDS-ADRDA	na	Low risk
Yamamoto 2002 [57]	177 (62.1%)		Patients with first-ever symptomatic lacunar infarct*	69.1 ± 8.6 (47–86)	D, ND	Dementia: Diagnostic and Statistical Manual of Mental Disorders, CI: CDR score 0.5 or HDSR score 21–26	CDR, HDS-R	Low risk

*N* number of patients, *NOS* Newcastle–Ottawa Scale, *D* dippers, *ED* extreme dippers, *ND* non-dippers, *RD* reverse dippers, *AD* Alzheimer disease, *VaD* vascular dementia, *MCI* mild cognitive impairment, *PDD* Parkinson’s disease with dementia, *na* non-applicable, *MMSE* Mini Mental State Examination, *FAB* Frontal Assessment Battery, *FR BMDB* Final Result of the Brief Mental Deterioration Battery, *JMCIS* Japanese version of the MCI screen, *CDR* Clinical Dementia Rating, *I-ADL* Instrumental Activities of Daily Living, *HDS-R* Hasegawa Dementia Scale-Revised, *MoCA* Montreal Cognitive Assessment, *SVAT* Simple Verbal Analogies Test, *RAVLT IR* Rey’s 15 words, immediate recall, *RAVLT DR* Rey’s 15 words: delayed recall, *IVM* Immediate visual memory, *FP* Verbal Phonemic Fluency, *FS* Verbal Semantic Fluency, *CD* Simple Copy Drawing, *PC* Pentagon Copy., *mHIS* modified Hachinski Ischemic Score, *NINDS-AIREN* National Institute for Neurological Disorders and Stroke-Association Internationale pour la Recherche et l’Enseignement en Neurosciences, *MDS* Movement Disorder Society, *NINCDS-ADRDA* National Institute of Neurological and Communicative Disorders and Stroke and the Alzheimer Disease and Related Disorders Association guidelines

*Admitted to the Department of Neurology at the Kyoto Second Red Cross Hospital between January 1987 and December 1991

^±^Admitted to the Department of Neurology at the Kyoto Second Red Cross Hospital between January 1997 and June 2000

^f^Admitted to the Department of Neurology at the Kyoto Second Red Cross Hospital between 2002 and 2005

**Table 2:** Summary of studies reporting on the association of circadian blood pressure patterns and performance in neuropsychological tests

Identity	*N* (% Males)		Population characteristics	Age (years)	Circadian blood pressure patterns	Global function	Attention/Working Memory	Memory	Executive function	Processing speed	NOS score
*Cross-sectional studies*											
Cicconetti 2003 [58]	40 (35%)		Newly diagnosed (< 1 year), never treated hypertensives without cerebrovascular history	62.9 ± 9.7	D, ND	MMSE	na	na	na	na	Fair
Cicconetti 2004 [59]	30 (10%)		Recently diagnosed (< 2 years), never treated hypertensives without cerebrovascular history	68.3 ± 7.3	D, ND	MMSE	na	na	na	na	Fair
Gregory 2016 [60]	115 (37%)		na	72 ± 7	D, ND	na	na	Immediate and delayed recall	TMT-A, TMT-B	DSC	High risk
Kececi 2016 [47]	91 (23%)		Aged ≥ 60 years hypertensive patients without comorbid diseases	71.9 ± 7.7	D, ND	sMMT	na	na	na	na	Fair
Khaled 2014	55 (50.9%)		Hypertensives without cerebrovascular, diabetes, renal disease or cardiovascular history	69.1 ± 3.6	D, ND	MMSE	na	na	na	na	Fair
Komori 2016 [49]	444 (61.5%)		Hospitalized heart failure patients without cardiovascular or cerebrovascular history	68 ± 13	D, ND, RD	MMSE	na	na	na	na	Low risk
Ohya 2001 [62]	99 (22%)		Elderly in nursing homes or geriatric hospitals	79.8 ± 10.1	D, ND, RD	MMSE	na	na	na	na	Fair
Sierra 2015 [63]	56 (66%)		Νever-treated essential hypertensive patients without cerebrovascular, diabetes, renal disease or cardiovascular history	54.3 ± 3.1	D, ND	na	Digit Span WAIS-R forward/backward	Russell Revision of the Logical Memory subscale, Visual Reproduction subscale of the Wechsler Memory Scale	na	na	High risk
Suzuki 2011 [67]	107 (30%)		Institutionalized dementia patients	76.3 ± 9.2	D, ND	MMSE	na	na	na	na	High risk
Tanaka 2018 [53]	137 (46%)		Parkinson’s disease patients	64.1 ± 10.5	D, ED, ND, RD	MMSE, HDS-R	na	na	na	na	Low Risk
White 2018 [40]	199 (45.7%)		Patients with qualitative evidence of WMH but without cerebrovascular, uncontrolled diabetes, renal disease or cardiovascular history	81.2 ± 4.1	D, ND	na	na	na	TMT-A, TMT-B, Stroop Color and Word Test	Simple Reaction Time from the California Computerized Assessment Package	Low risk
Yaneva-Sirakova 2016 [64]	439 (36.4%)		Hypertensives on combination therapy	64.7 ± 10.2	D, ND, RD	MMSE	na	na	na	na	High risk
*Prospective cohort studies*											
Ghazi 2020 [65]		1500 (56%)	NDD-CKD	63 ± 10	D, ED, ND, RD	3MS	na	na	na	na	High risk

*N* number of patients, *D* dippers, *ED* extreme dippers, *ND* non-dippers, *RD* reverse dippers, *MMSE* mini mental state examination, *sΜΜΤ* standardized mini mental test, *3MS* modified mini mental state examination, *TMT* Trail Making Tests, *DSC* Digit Symbol Coding, *WAIS-R* Wechsler Adult Intelligence Scale-Revised, *HDS-R* Hasegawa dementia scale-revised, *NDD-CKD* non–dialysis-dependent chronic kidney disease, *WMH* white matter hyperintensity

### Descriptive characteristics and risk of bias within studies

Eighteen studies (fifteen cross-sectional and three cohort studies) examined the risk of abnormal cognitive function among dipping groups in four thousand nine hundred and fifty-five patients (mean age 72.8 ± 9.1) [25–27, 44–57]. Ten studies reported on 2918 patients (mean age 65.6 ± 11.2) global function neuropsychological tests stratified by dipping patterns [40, 47, 49, 53, 58–65].

Nineteen out of twenty-seven eligible studies examining four thousand six hundred and forty-three patients reported on population’s hypertension history (Supplementary Table 3). 76.7% of the patients examined were hypertensives. Chen et al. was the only study investigating non-hypertensive patients [44]. Cani et al. examined the incidence of supine hypertension in a cohort of patients with idiopathic autonomic failure (IAF) [66]. Eight studies did not comment on hypertension history status of their population cohort [25, 27, 46, 51, 52, 60, 62, 67] while five [25, 27, 51, 52, 67] of them stated that some patients received antihypertensive treatment. Guo and Ohya et al. reported that none of the patients included received any antihypertensive treatment [46, 62].

Sierra et al. was the only study which focused on attention/working memory neuropsychological testing among dipping patterns using Digit Span Wechsler Adult Intelligence Scale-Revised forward/backward [63]. No difference was observed between dippers and non-dippers. Delayed memory [60, 63], executive function [40, 60], and processing speed [40, 60] were investigated in two studies each. Non-dippers performed worse than dippers in delayed memory tests as found by both Sierra and Gregory et al. [60, 63]. According to Gregory et al., non-dippers presented worse on executive function and processing speed assessments [60]. However, White et al. found no difference between the two groups in terms of these cognitive domains except for a slightly better performance of non-dippers in Trail Making Test Part A [40].

Overall, 13 studies were deemed of low risk of bias applying Newcastle–Ottawa Quality Assessment Scale, while 9 presented high risk of bias and 6 fair quality (Supplementary Table 5–6).

### Primary outcome: risk of cognitive impairment or dementia

#### Risk of abnormal cognitive function in dippers versus non-dippers

**Fig. 2:**
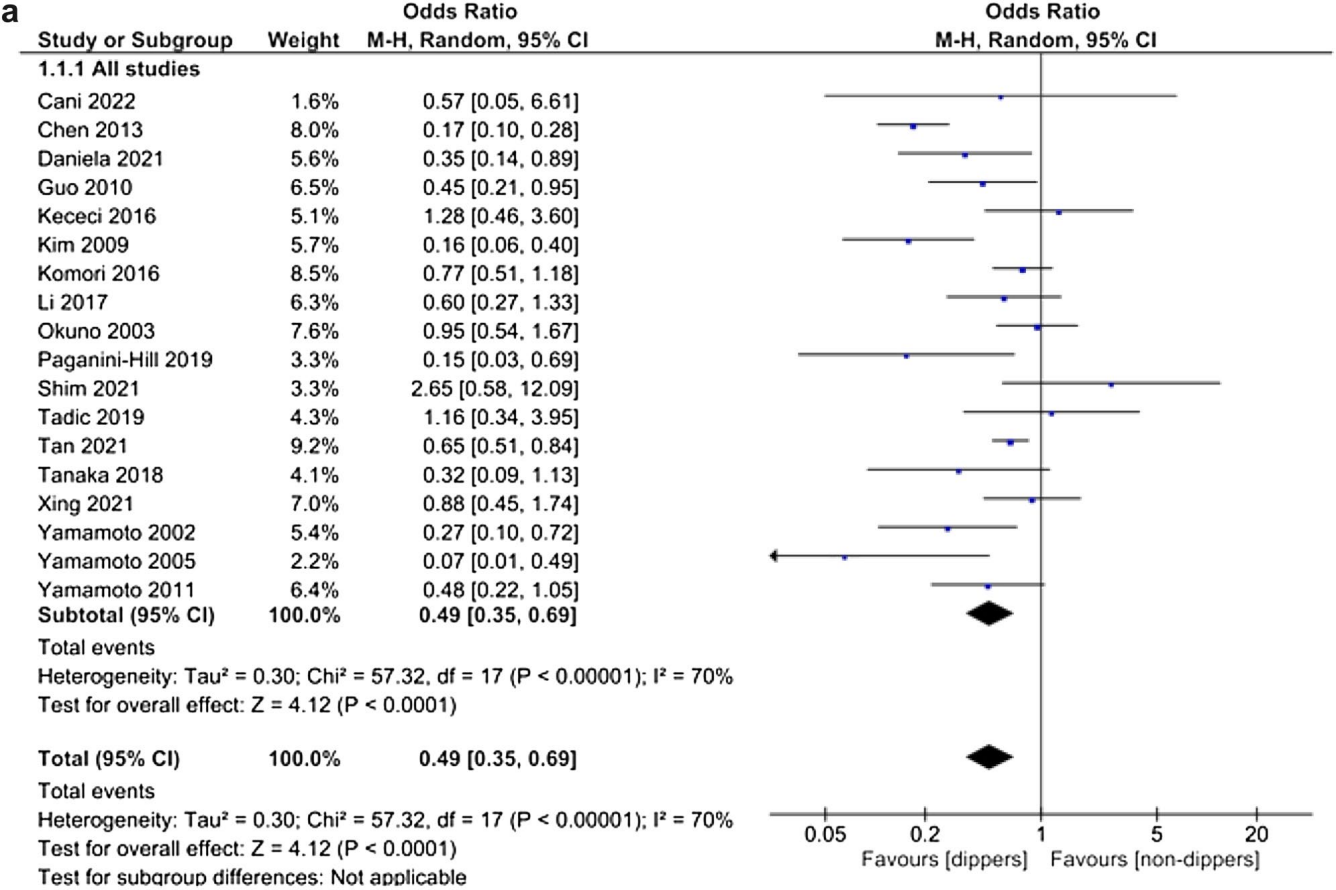

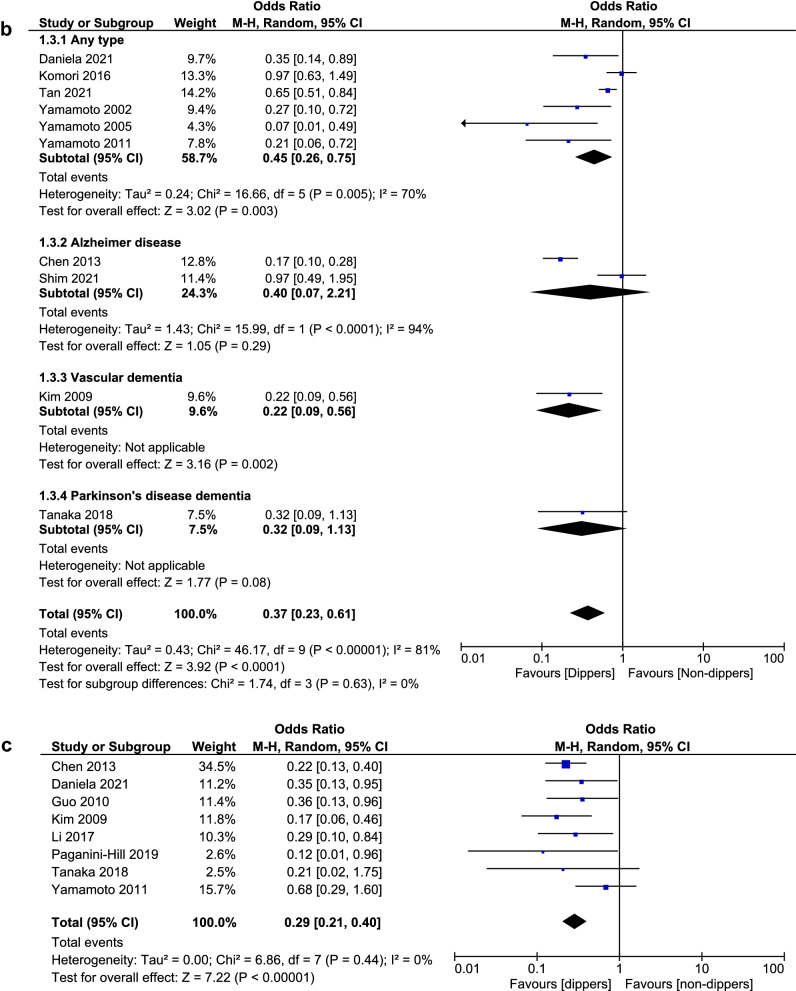
**A** Forest plot of pooled analysis on risk of abnormal cognitive function in dippers (including extreme dippers) versus non-dippers (including reverse dippers). **B** Forest plot of subgroup analysis on risk of dementia in dippers (including extreme dippers) versus non-dippers (including reverse dippers). **C** Forest plot of subgroup analysis on risk of abnormal cognitive function in dippers versus non-dippers

Pooled analysis of 18 studies examining 4740 patients indicated that dippers (dipping ≥ 10%, including extreme dippers depending on data availability) were 51% [OR 0.49 (95% CI 0.35–0.69), *I*^2^ 70%, Fig. 2A] less likely to present abnormal cognitive function than non-dippers (dipping < 10%, including reverse dippers depending on data availability) without any evidence of publication bias (Supplementary Fig. 1) but with significant between-study heterogeneity that remained in the sensitivity analysis (Supplementary Fig. 2). Thus, we performed the pre-planned subgroup analyses according to cognitive impairment subtypes in an effort to reduce heterogeneity. Risk of mild cognitive impairment was not statistically significantly different between dippers (dipping ≥ 10%) and non-dippers (dipping < 10%) [OR 0.84 (95% CI 0.55–1.27), *I*^2^ 36%, Supplementary Fig. 3]. Interestingly, pooled subgroup analysis of 9 studies with 3391 participants showed that dippers (dipping ≥ 10%) were 63% [OR 0.37 (95% CI 0.23–0.61), *I*^2^ 81%, Fig. 2B] less likely to present with dementia compared with non-dippers (dipping < 10%), with significant heterogeneity between studies.

Heterogeneity was eliminated when we compared dippers versus non-dippers without including extreme and reverse dippers, respectively (Fig. 2C). Pooled analysis of 8 studies with 887 participants indicated that dippers (dipping 10–20%) were 71% [OR 0.29 (95% CI 0.21–0.4), *I*^2^ 0%, Fig. 2C] less likely to present the primary outcome (cognitive impairment or dementia) than non-dippers (dipping 0–10%).

#### Risk of abnormal cognitive function in reverse dippers

**Fig. 3:**
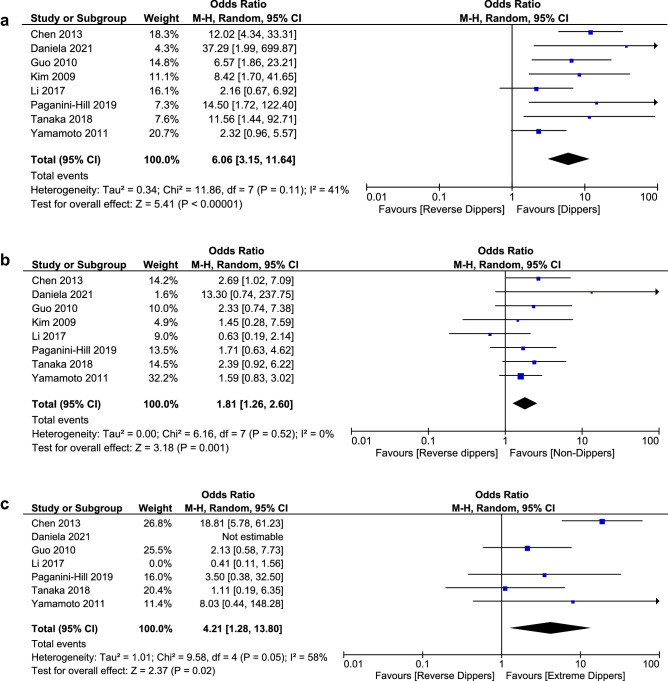
**A** Forest plot of subgroup analysis on risk of abnormal cognitive function in reverse dippers versus dippers. **B** Forest plot of subgroup analysis on risk of dementia in reverse dippers versus non-dippers. **C** Forest plot of sensitivity analysis on risk of abnormal cognitive function in reverse dippers versus extreme dippers

Pooled analysis of 8 studies with 611 participants suggested that the risk of abnormal cognitive function was 6 times higher in reverse dippers (dipping < 0%) than in dippers [OR 6.06 (95% CI 3.15–11.64), *I*^2^ 41%, Fig. 3A]. Reverse dippers also presented a higher risk of abnormal cognitive function than the rest of non-dippers (dipping 0–10%), as shown at Fig. 3B [OR 1.81 (95% CI 1.26–2.6), *I*^2^ 0%]. No statistically significant difference was observed in the risk of abnormal cognitive function between reverse and extreme dippers (dipping ≥ 20%) [OR 2.67 (95% CI 0.71–10.02), *I*^2^ 75%, Supplementary Fig. 4] but with evidence of substantial heterogeneity between studies. However, the sensitivity analysis revealed that reverse dippers had a statistically significant higher risk of abnormal cognitive function than extreme dippers [OR 4.21 (95% CI 1.28–13.8), *I*^2^ 58%, Fig. 3C].

#### Risk of abnormal cognitive function in extreme dippers

Extreme dippers (dipping ≥ 20%) did not have any statistically significant different risk of abnormal cognitive function compared with the rest of dippers (dipping 10–20%) [OR 2.52 (95% CI 0.92–6.94), *I*^2^ 58%, Supplementary Fig. 5] or non-dippers [OR 0.74 (95% CI 0.26–2.12), *I*^2^ 69%, Supplementary Fig. 6], respectively.

### Secondary outcome: cognitive function among dipping patterns

**Fig. 4:**
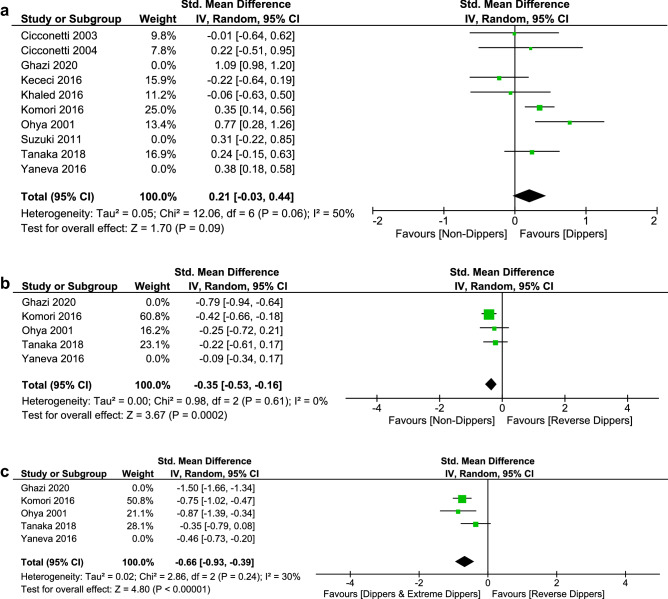
**A** Forest plot of sensitivity analysis on global function neuropsychological tests in dippers (including extreme dippers) versus non-dippers (including reverse dippers). **B** Forest plot of sensitivity analysis on global function neuropsychological tests in reverse dippers versus non-dippers. **C** Forest plot of sensitivity analysis on global function neuropsychological tests in reverse dippers versus dippers (including extreme dippers)

Pooled analysis of 10 studies with 2918 participants indicated that no statistically significant difference exists in global function neuropsychological tests [SMD 0.33 (95% CI − 0.01 to 0.67), *I*^2^ 92%, Supplementary Fig. 7] between dippers (dipping ≥ 10%, including extreme dippers depending on data availability) and non-dippers (dipping < 10%, including reverse dippers depending on data availability). Evidence of publication bias was suspected by visual inspection of funnel plot (Supplementary Fig. 8), and substantial heterogeneity between studies was detected. Sensitivity analysis reduced the heterogeneity and supported the previous result [SMD 0.21 (95% CI − 0.03 to 0.44), *I*^2^ 50%, Fig. 4A].

Interestingly, we found that reverse dippers (dipping < 0%) scored less in global function neuropsychological tests than both non-dippers (dipping 0–10%) [SMD − 0.35 (95% CI − 0.53 to − 0.16), *I*^2^ 0%, Fig. 4B] and dippers (dipping ≥ 10%, including extreme dippers depending on data availability) [SMD − 0.66 (95% CI − 0.93 to − 0.39), *I*^2^ 30%, Fig. 4C], respectively.

### Quality of evidence analysis and risk of bias

Quality of evidence was low for the primary and very low for the secondary outcome applying the GRADE tool (Supplementary Table 4) mainly due to observational design of the studies and the inconsistency across studies included in this meta‐analysis.

## Discussion

### Summary of evidence

The present meta-analysis of 7595 participants from 28 studies provides novel data regarding the association of disrupted circadian BP rhythms with abnormal cognitive function. The main findings of our study are as follows. First, when patients are dichotomized as ‘dippers’ and ‘non-dippers’, the normal dipping BP pattern is associated with a 51% lower risk of abnormal cognitive function (composite of cognitive impairment or dementia), and a 63% lower risk of dementia alone, compared to the non-dipping pattern. Results were reproduced in the pooled analysis excluding the two extreme subcategories (extreme dippers and reverse dippers), which rendered a 71% lower risk for abnormal cognitive function in dippers (dipping 10–20%) compared to non-dippers (dipping 0–10%). Second, reverse dipping presents the strongest association with abnormal cognitive function. Reverse dippers bear an up to sixfold higher risk of abnormal cognitive function compared to dippers, and an almost twofold higher risk compared to the rest of the non-dipping population. Reverse dippers also perform worse in global function neuropsychological tests compared with both dippers and the rest of non-dippers. Finally, extreme dipping does not appear to be associated with increased risk of abnormal cognitive function. These findings support that analyzing an individual’s dipping profile using 24 h ABPM might allow identification of individuals at higher risk for cognitive decline and dementia.

### Interpretation of the results in the context of other evidence

To the best of our knowledge, this is the first systematic review to provide meta-analytic data on the association of dipping patterns with cognitive impairment and dementia. The study results appear in line with a solid body of evidence demonstrating pronounced cardiac and extra-cardiac organ damage in populations with abolished normal circadian BP fall, specifically non-dippers and reverse dippers [17–19]. Recent systematic reviews and meta-analyses have demonstrated that nighttime dipping is a powerful predictor of cardiovascular and total mortality, total cardiovascular events (CVEs), coronary events, and when it comes to cerebrovascular outcomes, strokes as a common manifestation of hypertension-related brain damage [19, 68]. On the other hand, available information regarding extreme dipping remains inconclusive and controversial, with different reports suggesting similar, better, or worse cardiovascular prognosis as compared to dippers [69]. More recent evidence indicates that the association of extreme dipping with cardiovascular events strongly depends on age, with a U-shaped relationship of nocturnal BP dipping and adverse outcomes observed in subjects older than 70 years [70]. Different associations of dipping with varying outcomes in populations with divergent sample characteristics are indicative of the complexity of underlying mechanisms [71–73]. Results produced from our study on extreme dipping as compared to low-risk reference group (i.e., dippers) do not allow to conclude that a marked nocturnal BP fall is associated with cognitive decline and dementia. Still, the prognostic potential of extreme dipping in terms of brain-related outcomes needs to be verified in future studies.

Findings of our study provide further insight on potential underlying mechanisms and clinical implications. Several factors might account for the observed association of dipping patterns, specifically non-dipping (including or excluding reverse dipping) and reverse dipping alone, with abnormal cognitive function. Hypertension-induced mechanisms of brain damage have been described in detail elsewhere [8]. These include the induction of silent small vessel disease and cerebrovascular damage in the form of lacunae infarct and white mater lesions, which may substantially contribute to the subsequent development of clinically detectable cognitive impairment and vascular dementia [74]. It could be reasonably hypothesized that patients whose BP does not drop during nighttime according to the physiological circadian rhythm, therefore remaining at substantially increased levels throughout the whole 24 h period, are more susceptible to the detrimental effects of hypertension in the brain. This would be particularly true for reverse dippers, whose BP during the nighttime period even exceeds daytime BP levels. On the other hand, the disruption in the sleep–wake cycle is often encountered in patients with dementia and may disturb the normal circadian rhythm by turning a dipper into a non-dipper individual [75]. Longitudinal, appropriately designed studies are warranted to determine whether variations of the normal circadian BP drop precede cognitive decline, and if so, which pathophysiological mechanisms are implicated.

Regardless of the causality of the observed associations in our study, these may provide important clinical implications. It is being increasingly recognized that BP recording throughout a whole 24 h period offers much more information compared to conventional office BP measurements [31], and its use is being recommended in current hypertension guidelines as the method of choice for BP monitoring, whenever available. Therefore, physicians applying 24 h ABPM in the context of hypertension diagnosis and monitoring could identify patients at risk for cognitive decline (non-dippers and especially reverse dippers) and perform further diagnostic tests toward this direction or refer them to neurologists. Considering the wide applicability of 24 h ABPM, identification of patients at risk based on dipping patterns could unveil several cases of undiagnosed cognitive impairment, who would benefit the most from early interventions. Furthermore, provided that longitudinal studies confirm that abnormal dipping precedes cognitive dysfunction, the addition of ABPM to clinic-based BP could facilitate early interventions targeting modifiable risk factors at a pre-symptomatic stage before the clinical onset of cognitive impairment. Finally, further studies are warranted to investigate whether chronotherapy, aiming at restoring the nocturnal circadian BP rhythm in hypertensive patients, might decelerate the progression to clinically overt cognitive decline.

## Limitations

The limitations of this meta-analysis lie in the quality of the included studies. The observational design of studies retrieved from the literature downgraded our certainty of the evidence. Moreover, the studies had highly heterogeneous populations examining elderly patients with or without previous history of cardiovascular/cerebrovascular disease or antihypertensive therapy administration. However, the scarce data provided from existing studies limited our intention to perform pre-planned subgroup analyses, i.e. by age, history of hypertension/antihypertensive medication or cognitive domains studied. These factors may influence the cognitive status of the participants. The synthesis of these heterogeneous populations might have led to an underestimation of the overall association between dipping patterns and cognitive function. Another factor that downgraded the quality of evidence of this meta-analysis was the inconsistency of the results from population-based studies possibly due to the heterogeneous populations along with the different methods and the variations in the cognitive tests used to evaluate cognitive impairment and dementia among studies. All the above resulted in low certainty of evidence for the primary and very low for the secondary outcome when applying the GRADE tool. Nonetheless, this is the first meta-analysis examining the association between ABPM patterns and cognitive function. A pre-specified published protocol was strictly followed highlighting the methodological reliability and internal validity of this study.

## Perspectives

According to the present study, dysregulation of the normal circadian BP rhythm, i.e., non-dipping and reverse dipping, is associated with abnormal cognitive function (cognitive impairment and dementia). The addition of 24 h ABPM to clinic BP measurements might identify subjects at increased risk for cognitive impairment based on their individual dipping profile and facilitate timely diagnosis and early interventions. Future studies with a prospective design are needed to unveil the sequence of the observed associations, provide mechanistic insights and investigate potential benefits of preventive strategies targeting at restoring the physiological BP circadian rhythm in terms of cognitive function.

## Novelty and relevance

1. **What is new?**
2. The first systematic review and meta-analysis examining the association between ABPM patterns and cognitive function.
3. Dippers have a 63% lower risk for dementia compared to non-dippers.
4. Reverse dippers bear an up to sixfold higher risk of abnormal cognitive function compared to dippers.
5. **What is relevant?**
6. Dysregulation of the normal circadian BP rhythm, specifically non-dipping and reverse dipping is associated with abnormal cognitive function.
7. **Clinical implications?**
8. The addition of 24 h ABPM to clinic BP measurements might identify subjects at increased risk for cognitive impairment based on their individual dipping profile and facilitate timely diagnosis and early interventions.

## Supplementary Information

Below is the link to the electronic supplementary material.

Supplementary file1 (DOCX 441 KB)

## Data Availability

Raw data that support the findings of this study are available from the corresponding author, EG, upon reasonable request.
