# Supplementary material for: Association between ambulatory blood pressure monitoring patterns with cognitive function and risk of dementia: a systematic review and meta-analysis
Source: Aging Clin Exp Res. 2023 Mar 30;35(4):745–61. doi: 10.1007/s40520-023-02361-7 (PMC10115699; doi:10.1007/s40520-023-02361-7)

**Association between ambulatory blood pressure monitoring patterns with cognitive function and risk of dementia: a systematic review and meta-analysis**

Maria Gavriilaki ^1^, Panagiota Anyfanti ^2^, Konstantinos Mastrogiannis ^2^, Eleni Gavriilaki ^3^, Antonios Lazaridis ^2^, Vasilios K Kimiskidis ^1^, Eugenia Gkaliagkousi ^2^

^1^ 1st Department of Neurology, AHEPA University Hospital, School of Medicine, Aristotle University of Thessaloniki, Greece

^2^ 3rd Department of Internal Medicine, Papageorgiou Hospital, Aristotle University of Thessaloniki, Thessaloniki, Greece

^3^ Hematology Department, G. Papanicolaou Hospital, Thessaloniki, Greece.

# **Supplement**

Contents

[**Supplement** 2](#_Toc124369477)

[**Supplementary Material I. Supplementary Tables** 4](#_Toc124369478)

[**Supplementary Table 1: PRISMA 2020 checklist** 4](#_Toc124369479)

[**Supplementary Table 2: PRISMA abstract checklist** 7](#_Toc124369480)

[**Supplementary Table 3: Baseline population characteristics of included studies.** 8](#_Toc124369481)

[**Supplementary Table 4: GRADE evidence profile** 10](#_Toc124369482)

[**Supplementary Table 5: Newcastle-Ottawa scale (NOS) for included cross sectional studies** 11](#_Toc124369483)

[**Supplementary Table 6: Newcastle-Ottawa scale (NOS) for included cohort studies** 13](#_Toc124369484)

[**Supplementary Material II. Search strategy developed for each database** 14](#_Toc124369485)

[**Pubmed** 14](#_Toc124369486)

[**Cochrane** 14](#_Toc124369487)

[**EMBASE** 15](#_Toc124369488)

[**Supplementary Material III. Supplementary Figures** 17](#_Toc124369489)

[**Supplementary figure 1. Funnel plot of pooled analysis on risk of abnormal cognitive function in dippers versus non-dippers** 17](#_Toc124369490)

[**Supplementary figure 2. Forest plot of sensitivity analysis on risk of abnormal cognitive function in dippers versus non-dippers** 18](#_Toc124369491)

[**Supplementary figure 3. Forest plot of subgroup analysis on risk of mild cognitive impairment in dippers versus non-dippers** 19](#_Toc124369492)

[**Supplementary figure 4. Forest plot of pooled analysis on risk of abnormal cognitive function in reverse dippers versus extreme dippers** 20](#_Toc124369493)

[**Supplementary figure 5. Forest plot of pooled analysis on risk of abnormal cognitive function in extreme dippers versus dippers** 21](#_Toc124369494)

[**Supplementary figure 6. Forest plot of pooled analysis on risk of abnormal cognitive function in extreme dippers versus non-dippers** 22](#_Toc124369495)

[**Supplementary figure 7. Forest plot of pooled analysis on global function neuropsychological tests in dippers versus non-dippers** 23](#_Toc124369496)

[**Supplementary figure 8. Funnel plot of pooled analysis on global function neuropsychological tests in dippers versus non-dippers** 24](#_Toc124369497)

## **Supplementary Material I. Supplementary Tables**

### **Supplementary Table 1: PRISMA 2020 checklist**

| **Section and Topic** | **Item #** | **Checklist item** | **Page where item**  **is reported** |
| --- | --- | --- | --- |
| **TITLE** | | |  |
| Title | 1 | Identify the report as a systematic review. | 1 |
| **ABSTRACT** | | |  |
| Abstract | 2 | See the PRISMA 2020 for Abstracts checklist. | 2 |
| **INTRODUCTION** | | |  |
| Rationale | 3 | Describe the rationale for the review in the context of existing knowledge. | 4,5 |
| Objectives | 4 | Provide an explicit statement of the objective(s) or question(s) the review addresses. | 5 |
| **METHODS** | | |  |
| Eligibility criteria | 5 | Specify the inclusion and exclusion criteria for the review and how studies were grouped for the syntheses. | 6 |
| Information sources | 6 | Specify all databases, registers, websites, organisations, reference lists and other sources searched or consulted to identify studies. Specify the date when each source was last searched or consulted. | 6 |
| Search strategy | 7 | Present the full search strategies for all databases, registers and websites, including any filters and limits used. | PROSPERO database (ID: CRD42022310384) |
| Selection process | 8 | Specify the methods used to decide whether a study met the inclusion criteria of the review, including how many reviewers screened each record and each report retrieved, whether they worked independently, and if applicable, details of automation tools used in the process. | 7 |
| Data collection process | 9 | Specify the methods used to collect data from reports, including how many reviewers collected data from each report, whether they worked independently, any processes for obtaining or confirming data from study investigators, and if applicable, details of automation tools used in the process. | 7 |
| Data items | 10a | List and define all outcomes for which data were sought. Specify whether all results that were compatible with each outcome domain in each study were sought (e.g. for all measures, time points, analyses), and if not, the methods used to decide which results to collect. | 7 |
|  | 10b | List and define all other variables for which data were sought (e.g. participant and intervention characteristics, funding sources). Describe any assumptions made about any missing or unclear information. | 7 |
| Study risk of bias assessment | 11 | Specify the methods used to assess risk of bias in the included studies, including details of the tool(s) used, how many reviewers assessed each study and whether they worked independently, and if applicable, details of automation tools used in the process. | 7 |
| Effect measures | 12 | Specify for each outcome the effect measure(s) (e.g. risk ratio, mean difference) used in the synthesis or presentation of results. | 7,8 |
| Synthesis methods | 13a | Describe the processes used to decide which studies were eligible for each synthesis (e.g. tabulating the study intervention characteristics and comparing against the planned groups for each synthesis (item #5)). | 8 |
|  | 13b | Describe any methods required to prepare the data for presentation or synthesis, such as handling of missing summary statistics, or data conversions. | na |
|  | 13c | Describe any methods used to tabulate or visually display results of individual studies and syntheses. | 8 |
|  | 13d | Describe any methods used to synthesize results and provide a rationale for the choice(s). If meta-analysis was performed, describe the model(s), method(s) to identify the presence and extent of statistical heterogeneity, and software package(s) used. | 8 |
|  | 13e | Describe any methods used to explore possible causes of heterogeneity among study results (e.g. subgroup analysis, meta-regression). | 8 |
|  | 13f | Describe any sensitivity analyses conducted to assess robustness of the synthesized results. | 8 |
| Reporting bias assessment | 14 | Describe any methods used to assess risk of bias due to missing results in a synthesis (arising from reporting biases). | na |
| Certainty assessment | 15 | Describe any methods used to assess certainty (or confidence) in the body of evidence for an outcome. | 8 |
| **RESULTS** | | |  |
| Study selection | 16a | Describe the results of the search and selection process, from the number of records identified in the search to the number of studies included in the review, ideally using a flow diagram. | 8 |
|  | 16b | Cite studies that might appear to meet the inclusion criteria, but which were excluded, and explain why they were excluded. | 9 |
| Study characteristics | 17 | Cite each included study and present its characteristics. | 9, tables |
| Risk of bias in studies | 18 | Present assessments of risk of bias for each included study. | 9 |
| Results of individual studies | 19 | For all outcomes, present, for each study: (a) summary statistics for each group (where appropriate) and (b) an effect estimate and its precision (e.g. confidence/credible interval), ideally using structured tables or plots. | figures 2-4 |
| Results of syntheses | 20a | For each synthesis, briefly summarise the characteristics and risk of bias among contributing studies. | tables |
|  | 20b | Present results of all statistical syntheses conducted. If meta-analysis was done, present for each the summary estimate and its precision (e.g. confidence/credible interval) and measures of statistical heterogeneity. If comparing groups, describe the direction of the effect. | 9-11 |
|  | 20c | Present results of all investigations of possible causes of heterogeneity among study results. | 9-11 |
|  | 20d | Present results of all sensitivity analyses conducted to assess the robustness of the synthesized results. | 9-11 |
| Reporting biases | 21 | Present assessments of risk of bias due to missing results (arising from reporting biases) for each synthesis assessed. | na |
| Certainty of evidence | 22 | Present assessments of certainty (or confidence) in the body of evidence for each outcome assessed. | 12 |
| **DISCUSSION** | | |  |
| Discussion | 23a | Provide a general interpretation of the results in the context of other evidence. | 13-15 |
|  | 23b | Discuss any limitations of the evidence included in the review. | 15 |
|  | 23c | Discuss any limitations of the review processes used. | 16 |
|  | 23d | Discuss implications of the results for practice, policy, and future research. | 16 |
| **OTHER INFORMATION** | | |  |
| Registration and protocol | 24a | Provide registration information for the review, including register name and registration number, or state that the review was not registered. | 6 |
|  | 24b | Indicate where the review protocol can be accessed, or state that a protocol was not prepared. | 6 |
|  | 24c | Describe and explain any amendments to information provided at registration or in the protocol. | na |
| Support | 25 | Describe sources of financial or non-financial support for the review, and the role of the funders or sponsors in the review. | 17 |
| Competing interests | 26 | Declare any competing interests of review authors. | 17 |
| Availability of data, code and other materials | 27 | Report which of the following are publicly available and where they can be found: template data collection forms; data extracted from included studies; data used for all analyses; analytic code; any other materials used in the review. | 17 |

### **Supplementary Table 2: PRISMA abstract checklist**

| **Section and Topic** | **Item #** | **Checklist item** | **Reported (Yes/No)** |
| --- | --- | --- | --- |
| **TITLE** | | |  |
| Title | 1 | Identify the report as a systematic review. | Yes |
| **BACKGROUND** | | |  |
| Objectives | 2 | Provide an explicit statement of the main objective(s) or question(s) the review addresses. | Yes |
| **METHODS** | | |  |
| Eligibility criteria | 3 | Specify the inclusion and exclusion criteria for the review. | Yes |
| Information sources | 4 | Specify the information sources (e.g. databases, registers) used to identify studies and the date when each was last searched. | Yes |
| Risk of bias | 5 | Specify the methods used to assess risk of bias in the included studies. | Yes |
| Synthesis of results | 6 | Specify the methods used to present and synthesis results. | Yes |
| **RESULTS** | | |  |
| Included studies | 7 | Give the total number of included studies and participants and summarise relevant characteristics of studies. | Yes |
| Synthesis of results | 8 | Present results for main outcomes, preferably indicating the number of included studies and participants for each. If meta-analysis was done, report the summary estimate and confidence/credible interval. If comparing groups, indicate the direction of the effect (i.e. which group is favoured). | Yes |
| **DISCUSSION** | | |  |
| Limitations of evidence | 9 | Provide a brief summary of the limitations of the evidence included in the review (e.g. study risk of bias, inconsistency and imprecision). | Yes |
| Interpretation | 10 | Provide a general interpretation of the results and important implications. | Yes |
| **OTHER** | | |  |
| Funding | 11 | Specify the primary source of funding for the review. | Yes |
| Registration | 12 | Provide the register name and registration number. | Yes |

### **Supplementary Table 3: Baseline population characteristics of included studies.**

| Identity | N_ examined | Hypertension, N (%) | Antihypertensive therapy, N (%) | Stroke, N (%) | Diabetes, N (%) | Renal disease, N (%) | Cardiovascular disease, N (%) | Hyperlipidemia, N (%) | Smoking, N (%) |
| --- | --- | --- | --- | --- | --- | --- | --- | --- | --- |
| Chen | 318 | 0 | 0 | 0 | na | 0 | 0 | na | na |
| Cicconetti 2003 | 40 | 40 (100) | 0 | 0 | 0 | 0 | 0 | 0 | 0 |
| Cicconetti 2004 | 30 | 30 (100) | 0 | 0 | 0 | 0 | 0 | 0 | 0 |
| Daniela | 90 | 39 (43.3) | 39(43.3) | 0 | 21(23.3) | na | na | na | na |
| Ghazi | 1502 | 1397 (93) | 1374(92) | 174(12) | 629(42) | 1132(75)na |  | 1027(69) | 131(9) |
| Gregory | 115 | na | na | na | na | na | na | na | na |
| Guo | 144 | na | 0 | 0 | 16(11.1) | na | 0 | na | na |
| Kececi | 91 | 91 (100) | 91(100) | 0 | 0 | 0 | 0 | 0 | na |
| Khaled | 55 | 55 (100) | na | na | 0 | na | na | 20(25.9) | 40(51.9) |
| Kim | 109 | 26 (70) | 22(60) | na | 13 (35) | na | na | 11(30) | na |
| Komori | 444 | 305 (68.69) | na | 0 | 154 (34.68) | 0 | 138(31.08) | na | na |
| vonkanel/Li | 108 | 29 (26.85) | na | na | 25 (23.14) | na | na | na | 39(36.1) |
| Ohya | 99 | na | 0 | 51(52) | 17(17) | na | 7(7) | na | na |
| Okuno | 204 | na | 126(61.7) | 0 | na | na | na | na | na |
| Paganini-Hill | 121 | 77 (64) | na | 11(9) | 14(12) | na | 19(16) | na | 56(46) |
| Shim | 174 | na | na | na | 50(28.7) | na | na | 93(53.4) | 42(24.1) |
| Sierra | 56 | 56 (100) | 0 | 0 | 0 | 0 | 0 | na | na |
| Suzuki | 107 | na | 45(42) | 79(73.8) | 3(2.8) | na | 21(19.6) | na | na |
| Tadic | 471 | na | 148(31.4) | na | na | na | na | na | 85(18) |
| Tan | 1608 | na | 600(37.3) | na | 436(27.1) | na | na | 560(34.8) | 243(15.1) |
| Tanaka | 137 | 29 (21.1) | 25(18.2) | 5 (3.7) | 14 (10.2) | na | 4 (3) | 25 (18.2) | 10 (7.3) |
| White | 199 | 199 (100) | na | 0 | na | na | 0 | na | na |
| Xing | 305 | 242 (79) | na | na | 120(39.3) | na | na | 151(46.5) | 31(10.2) |
| Yamamoto 2002 | 177 | 151 (85.3) | 0 | 177(100) | 25(14,1) | na | 46(26) | 51(28,8) | 39(22) |
| Yamamoto 2005 | 200 | 176 (88) | 0 | 200(100) | 23(11.5) | na | na | 75(37.5) | 45(22.5) |
| Yamamoto | 224 | 182 (81.25) | 0 | 224(100) | 36(16,1) | na | na | 61 (27.2) | 54(24,1) |
| Yaneva-Sirakova | 439 | 439 (100) | 439(100) | na | na | na | na | na | na |
| Cani | 30 | 21 (70) * | na | 0 | 1 (3) | na | 3 (10) | na | 2 (7) |

na: non-applicable, N: number of patients, *Supine hypertension

### **Supplementary Table 4: GRADE evidence profile**

| **Cognitive function in dippers compared to non-dippers** | | | | | |
| --- | --- | --- | --- | --- | --- |
|  | | | | | |
| **Outcomes** | **№ of participants (studies)** | **Certainty of the evidence (GRADE)** | **Relative effect (95% CI)** | **Anticipated absolute effects** | |
|  |  |  |  | **Risk with non-dippers** | **Risk difference with dippers** |
| Risk of abnormal cognitive function assessed with: OR | 4740 (18 observational studies) | ⨁⨁◯◯ Low | **OR 0.49** (0.35 to 0.69) | 387 per 1.000 | **151 fewer per 1.000** (206 fewer to 84 fewer) |
| Global function neuropsychological tests  assessed with: SMD | 2918 (10 observational studies) | ⨁◯◯◯ Very low | - | - | SMD **0.33 SD higher** (0.01 lower to 0.67 higher) |
| ***The risk in the intervention group** (and its 95% confidence interval) is based on the assumed risk in the comparison group and the **relative effect** of the intervention (and its 95% CI). **CI:** confidence interval; **OR:** odds ratio; **SMD:** standardised mean difference | | | | | |
| **GRADE Working Group grades of evidence** **High certainty:** we are very confident that the true effect lies close to that of the estimate of the effect. **Moderate certainty:** we are moderately confident in the effect estimate: the true effect is likely to be close to the estimate of the effect, but there is a possibility that it is substantially different. **Low certainty:** our confidence in the effect estimate is limited: the true effect may be substantially different from the estimate of the effect. **Very low certainty:** we have very little confidence in the effect estimate: the true effect is likely to be substantially different from the estimate of effect. | | | | | |

### **Supplementary Table 5: Newcastle-Ottawa scale (NOS) for included cross sectional studies**

| Cross sectional and Longitudal studies | | | | | | | | | |
| --- | --- | --- | --- | --- | --- | --- | --- | --- | --- |
|  | **selection** | | | | **Comparability** | **Exposure** | | **overall** | **NOS scale** |
| Study | **Representativeness of the sample** | **Sample size** | **Non-respondents** | **Ascertainment of the exposure (risk factor)** | **The subjects in different outcome groups are comparable, based on the study design or analysis. Confounding factors are controlled** | **Assessment of the outcome** | **Statistical test** |  |  |
| Chen | c | ***** | ***** | ****** | ** | d | * | 7 | Low Risk |
| Cicconetti 2003 | c | b | ***** | ****** | ** | d | * | 6 | Fair |
| Cicconetti 2004 | c | b | ***** | ****** | ** | d | * | 6 | Fair |
| Daniela | c | b | b | ****** | ** | d | * | 5 | High Risk |
| Gregory | d | ***** | b | **c** |  | d | * | 2 | High Risk |
| Guo | * | ***** | ***** | ****** | ** | d | * | 8 | Low Risk |
| Kececi | c | b | ***** | ****** | ** | d | * | 6 | Fair |
| Khaled | c | b | ***** | ****** | ** | d | * | 6 | Fair |
| Kim | * | ***** | ***** | ****** | ** | d | * | 8 | Low Risk |
| Komori | c | ***** | ***** | ****** | ** | d | * | 7 | Low Risk |
| vonkanel/Li | c | ***** | ***** | ****** |  | d | * | 5 | High Risk |
| Ohya | c | b | ***** | ****** | ** | d | * | 6 | Fair |
| Okuno | c | ***** | ***** | ****** | ** | ** | * | 9 | Low Risk |
| Paganini - Hill | c | ***** | ***** | ****** | ** | ** | * | 9 | Low Risk |
| Shim | c | ***** | ***** | ****** | ** | ** | * | 9 | Low Risk |
| Sierra | c | b | ***** | ****** | ** | d | no | 5 | High Risk |
| Suzuki | c | ***** | **b** | ****** |  | d | * | 4 | High Risk |
| Tadic | * | ***** | ***** | ****** |  | d | * | 6 | High risk |
| Tan | * | ***** | ***** | ****** | ** | ** | * | 10 | Low Risk |
| Tanaka | c | ***** | ***** | ****** | ** | d | * | 7 | Low Risk |
| White 2018 | c | ***** | ***** | ****** | ** | d | * | 7 | Low Risk |
| Xing | c | ***** | ***** | ****** |  | ** | * | 7 | High Risk |
| Yamamoto 2005 | c | ***** | ***** | ****** | ** | d | * | 7 | Low Risk |
| Yamamoto 2011 | c | ***** | ***** | ****** | ** | d | * | 7 | Low Risk |
| Yaneva-Sirakova | c | ***** | ***** | ****** |  | d | no | 4 | High risk |

### **Supplementary Table 6: Newcastle-Ottawa scale (NOS) for included cohort studies**

| Cohort studies | | | | | | | | | | | |
| --- | --- | --- | --- | --- | --- | --- | --- | --- | --- | --- | --- |
|  | | **Selection** | | | | **Comparability** | **Outcome** | | | **overall** | **NOS scale** |
|  | **Representativeness of the exposed cohort** | | **Selection of the non exposed cohort** | **Ascertainment of exposure** | **Demonstration that outcome of interest was not present at start of study** |  | **Assessment of outcome** | **Was follow-up long enough for outcomes to occur** | **Adequacy of follow up of cohorts** |  |  |
| Cani | c | | * | * | b | ** | * | * | * | 7 | Fair |
| Ghazi | c | | * | * | b | ** | d | * | d | 5 | High risk |
| Yamamoto 2002 | c | | * | * | * | ** | * | * | d | 7 | Low Risk |

## **Supplementary Material II. Search strategy developed for each database**

### **Pubmed**

Search: ((((((((((((((dipping[Text Word]) OR (nocturnal hypertension[Text Word])) OR (dipper[Text Word])) OR (dipper hypertension[Text Word])) OR (nondipper[Text Word])) OR (non-dipper[Text Word])) OR (nocturnal dipping[Text Word])) OR (nightime blood pressure[Text Word])) OR (circadian blood pressure[Text Word])) OR (nocturnal blood pressure[Text Word])) OR (dipper*[Text Word])) OR (non-dipp*[Text Word])) OR (nondipp*[Text Word])) OR ((((("Blood Pressure Monitoring, Ambulatory"[Mesh]) OR ('ambulatory blood pressure'[Text Word])) OR (ambulatory blood pressure measurement[Text Word])) OR (ambulatory blood pressure monitoring[Text Word])) OR (abpm[Text Word]))) AND (((((((((((((((((("Memory Disorders"[Mesh]) OR ("Mental Status and Dementia Tests"[Mesh])) OR ("Cognitive Dysfunction"[Mesh])) OR ("Dementia"[Mesh])) OR (cognitive imparment[Text Word])) OR (dementia[Text Word])) OR (cognitive function[Text Word])) OR (cognitive defect[Text Word])) OR (cognitive decline[Text Word])) OR (MCI[Text Word])) OR (MMSE[Text Word])) OR (cognitive testing[Text Word])) OR (MOCA[Text Word])) OR (neuropsychological testing[Text Word])) OR (montreal cognitive assessment[Text Word])) OR (small vessel disease[Text Word])) OR (alzheimer disease[Text Word])) OR (mini mental state examination[Text Word]))

### **Cochrane**

#1 ('Blood Pressure Monitoring' OR 'ambulatory blood pressure' OR 'ambulatory blood pressure measurement' OR 'ambulatory blood pressure monitoring' OR ABPM):ti,ab,kw (Word variations have been searched) 21854

#2 MeSH descriptor: [Blood Pressure Monitoring, Ambulatory] explode all trees 1457

#3 #1 OR #2 21854

#4 ('dipping' OR 'nocturnal hypertension' OR 'dipper' OR 'dipper hypertension' OR 'nondipper' OR 'nocturnal dipping' OR 'nightime blood pressure' OR 'circadian blood pressure' OR 'nocturnal blood pressure' OR dipper* OR non-dipp* OR nondipp*):ti,ab,kw 2102

#5 MeSH descriptor: [Memory Disorders] explode all trees 1173

#6 MeSH descriptor: [Mental Status and Dementia Tests] explode all trees 552

#7 MeSH descriptor: [Cognitive Dysfunction] explode all trees 1491

#8 MeSH descriptor: [Dementia] explode all trees 5935

#9 ('cognitive imparment' OR 'cognitive function' OR 'cognitive defect' OR 'cognitive decline' OR 'dementia' OR 'cognitive dysfunction' OR 'memory disorders' OR 'mental status tests' OR 'vascular dementia' OR 'small vessel disease' OR 'alzheimer disease' OR 'mini mental state examination' OR MCI OR AD OR MMSE OR 'cognitive testing' OR 'moca' OR 'neuropsychological testing' OR 'montreal cognitive assessment'):ti,ab,kw 62606

#10 #5 OR #7 OR #6 OR #9 OR #8 63113

#11 #3 AND #4 AND #10 27

### **EMBASE**

#11 #9 AND #10

#10 #4 AND #5

#9 #6 OR #7 OR #8

#8 ‘vascular dementia’/exp OR ‘vascular dementia’ OR ‘small vessel disease’/exp OR ‘small vessel disease’ OR ‘alzheimer disease’/exp OR ‘alzheimer disease’ OR ‘alzheimer s disease assessment scale cognitive subscale’/exp OR ‘alzheimer s disease assessment scale cognitive subscale’ OR ‘mini mental state examination’/exp OR ‘mini mental state examination’

#7 ‘mci’ OR ‘ad’ OR ‘mmse’/exp OR ‘mmse’ OR ‘cognitive testing’ OR ‘moca’ OR ‘neuropsychological testing’ OR ‘montreal cognitive assessment’/exp OR ‘montreal cognitive assessment’

#6 ‘cognitive impairment’ OR ‘dementia’/exp OR ‘dementia’ OR ‘cognitive function’/exp OR ‘cognitive function’ OR ‘cognitive defect’/exp OR ‘cognitive defect’ OR ‘cognitive decline’/exp OR ‘cognitive decline’

#5 ‘dipping’ OR ‘nocturnal hypertension’/exp OR ‘nocturnal hypertension’ OR ‘dipper’ OR ‘dipper hypertension’/exp OR ‘dipper hypertension’ OR ‘non dipper’ OR ‘nocturnal dipping’ OR ‘nightime blood pressure’ OR ‘circadian blood pressure’ OR ‘nocturnal blood pressure’/exp OR ‘nocturnal blood pressure’

#4 #1 OR #2 OR #3

#3 ‘ambulatory bp levels’ OR ‘ambulatory blood pressure’/exp OR ‘ambulatory blood pressure’ OR ‘ambulatory blood pressure measurement’/exp OR ‘ambulatory blood pressure measurement’

#2 ‘ambulatory blood pressure monitoring’/exp OR ‘ambulatory blood pressure monitoring’ OR ’24-h blood pressure monitoring’ OR ‘abpm’

#1 ‘Blood pressure monitoring’/exp OR ‘blood pressure monitoring’

## **Supplementary Material III. Supplementary Figures**

### **Supplementary figure 1. Funnel plot of pooled analysis on risk of abnormal cognitive function in dippers versus non-dippers**

**
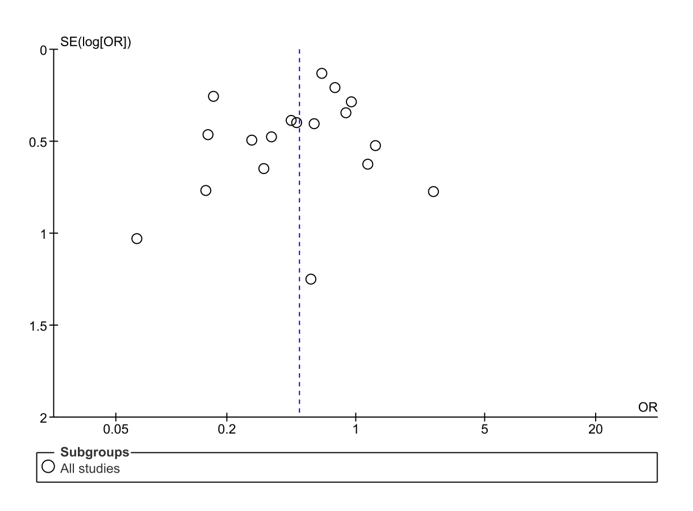
**

### **Supplementary figure 2. Forest plot of sensitivity analysis on risk of abnormal cognitive function in dippers versus non-dippers**

**
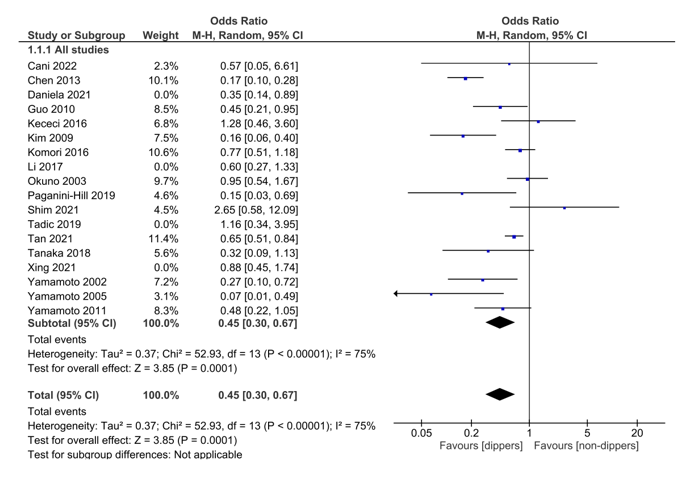
**

### **Supplementary figure 3.** **Forest plot of subgroup analysis on risk of mild cognitive impairment in dippers versus non-dippers**


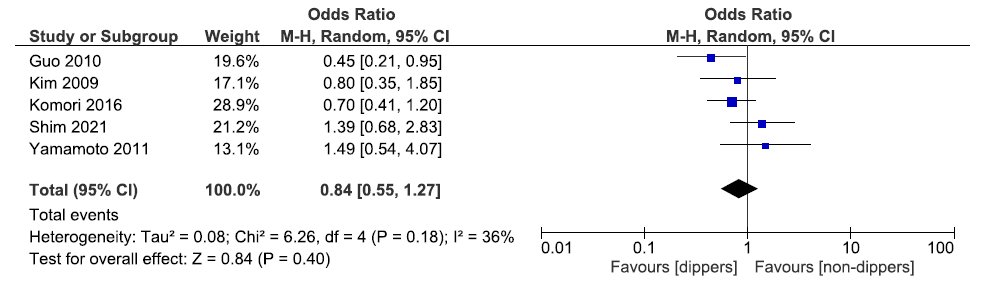


### **Supplementary figure 4. Forest plot of pooled analysis on risk of abnormal cognitive function in reverse dippers versus extreme dippers**


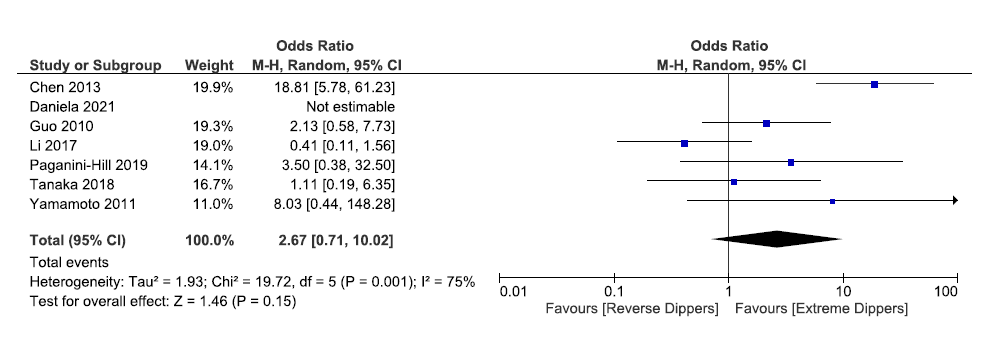


### **Supplementary figure 5. Forest plot of pooled analysis on risk of abnormal cognitive function in extreme dippers versus dippers**


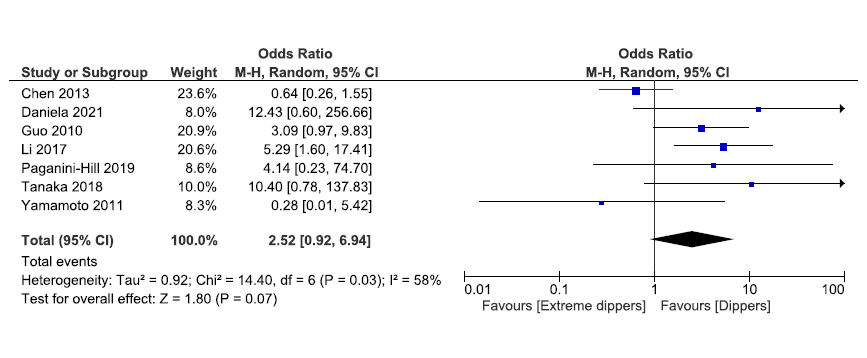


### **Supplementary figure 6. Forest plot of pooled analysis on risk of abnormal cognitive function in extreme dippers versus non-dippers**


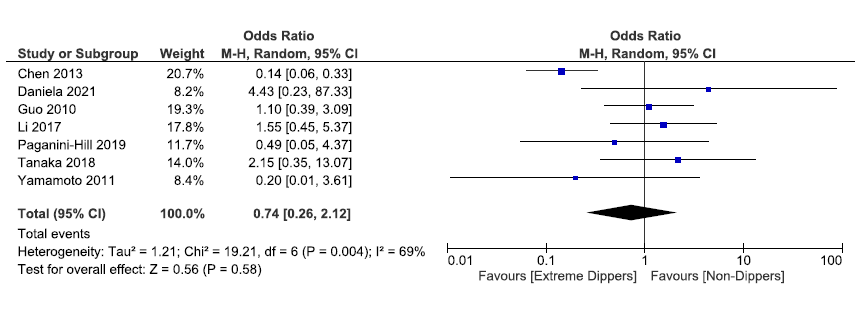


### **Supplementary figure 7. Forest plot of pooled analysis on global function neuropsychological tests in dippers versus non-dippers**


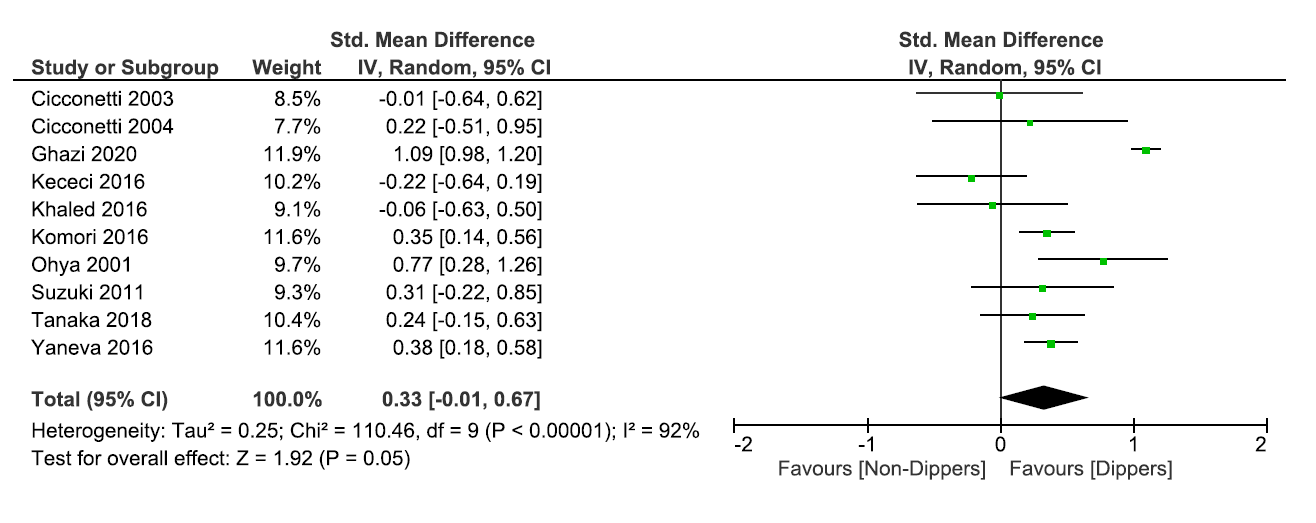


### **Supplementary figure 8. Funnel plot of pooled analysis on** **global function neuropsychological tests in dippers versus non-dippers**


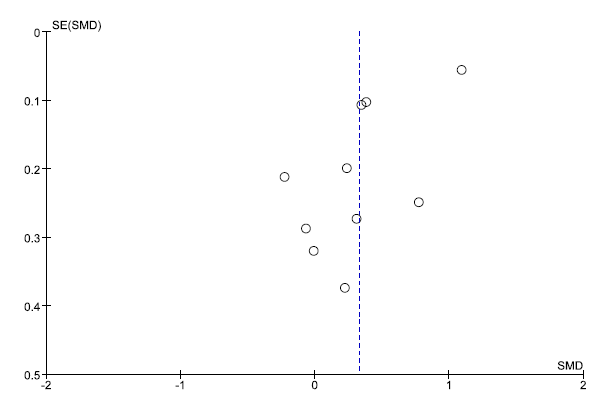

Supplement: Supplementary file 1 — Supplementary file1 (DOCX 441 KB) [file 40520_2023_2361_MOESM1_ESM.docx]
